# Bone-Specific Alkaline Phosphatase Levels among Patients with Multiple Myeloma Receiving Various Therapy Options

**DOI:** 10.4274/tjh.2013.0004

**Published:** 2014-12-05

**Authors:** Güven Çetin, Ahmet Emre Eşkazan, M. Cem Ar, Şeniz Öngören Aydın, Burhan Ferhanoğlu, Teoman Soysal, Zafer Başlar, Yıldız Aydın

**Affiliations:** 1 Bezmialem Vakıf University Faculty of Medicine, Department of Internal Medicine, Division of Hematology, İstanbul, Turkey; 2 Diyarbakır Training and Research Hospital, Clinic of Hematology, Diyarbakır, Turkey; 3 İstanbul Training and Research Hospital, Clinic of Hematology, İstanbul, Turkey; 4 İstanbul University Cerrahpaşa Faculty of Medicine, Department of Internal Medicine, Division of Hematology, İstanbul, Turkey

**Keywords:** Multiple myeloma, Bone-specific alkaline phosphatase, Bortezomib, Thalidomide

## Abstract

**Objective:** This study aimed to investigate the impact of the different therapy regimens used in multiple myeloma (MM) on bone-specific alkaline phosphatase (BALP) levels.

**Materials and Methods:** One hundred and thirteen patients with MM were included in the study. Patients were grouped according to the regimens they received, as follows: group 1, melphalan and prednisolone (MP); group 2, vincristine, adriablastin, and dexamethasone (VAD); group 3, thalidomide plus dexamethasone; and group 4, bortezomib plus dexamethasone. BALP levels were measured before treatment and at the third and sixth months of treatment. A fifth group consisted of patients in the post-treatment remission period at study entry (no-treatment group).

**Results:** The BALP levels at the third and sixth months of the treatment were significantly higher than the pre-treatment levels in the bortezomib and the no-treatment groups, whereas no significant difference was observed in the MP, VAD, and thalidomide groups.

**Conclusion:** Considering that BALP is a surrogate marker of bone formation, our study suggests that bortezomib more efficiently leads to the improvement of bone disease in myeloma than other treatment options.

## Abstract (tr)

**Amaç:** Bu çalışmanın amacı; multipl myelomda (MM) kullanılan farklı tedavi rejimlerinin kemik-spesifik alkalen fosfataz (BALP) düzeyleri üzerine etkisini incelemektir.

**Gereç ve Yöntemler:** Çalışmaya 113 MM hastası dahil edildi. Hastalar aldıkları tedavi rejimlerine göre; 1. grup, melfalan ve prednizolon (MP); 2. grup, vinkristin, adriablastin ve deksametazon (VAD); 3. grup talidomid artı deksametazon; 4. grup, bortezomib artı deksametazon olarak gruplara ayrıldı. BALP düzeyleri tedaviden önce, tedavinin üçüncü ve altıncı aylarında ölçüldü. Çalışmanın başında, tedavi sonrası remisyon dönemindeki hastalardan beşinci bir grup oluşturuldu (tedavi almayan grup).

**Bulgular:** Bortezomib grubu ve tedavi almayan grupta tedavinin üçüncü ve altıncı aylarında bakılan BALP düzeyleri, tedavi öncesine göre anlamlı olarak yüksek bulunurken; MP, VAD, ve talidomid gruplarında anlamlı fark saptanmadı.

**Sonuç:** BALP’nin kemik formasyonunun bir belirteci olduğu göz önünde tutulduğunda, bu çalışma ile diğer tedavi seçeneklerine göre bortezomib tedavisinin miyelomda kemik hastalığının iyileşmesine daha etkin bir şekilde yol açtığı gösterilmiştir.

## INTRODUCTION

Multiple myeloma (MM) is a malignant disease characterized by anemia, monoclonal protein in the serum and/or the urine, osteolytic bone lesions, hypercalcemia, and renal insufficiency resulting from uncontrollable clonal hyperproliferation of plasma cells in bone marrow [1,2,3]. The skeletal system problems that appear either at the time of the diagnosis or during the progression of MM cause a decrease in the quality of life and lead patients to present to health services. The rate of skeletal system-related complications including bone pain, osteolytic lesions, and pathological fractures reaches up to 80% in patients with MM [4,5]. In the studies concerning MM, it has been demonstrated that there is a significant relation between the bone-specific alkaline phosphatase (BALP) levels and bone pain, lytic lesions, and bone fractures [1,2]. 

Until now, there has been no large study showing the effect of various therapy options used in the treatment of MM on the changes in bone metabolism caused by myeloma. The aim of the present study was to investigate the relationship between pre-treatment and post-treatment BALP levels and the therapy options in patients with MM, and to expose the effect of these therapy options on bone metabolism.

## MATERIALS AND METHODS

**Patients**

One hundred and thirteen patients diagnosed with MM in accordance with the criteria of the International Myeloma Working Group and followed in the Hematology Division of the Internal Medicine Department of the İstanbul University Cerrahpaşa Medical Faculty between November 2006 and July 2009 were included in the present study. The study cohort consists of patients with MM that have been followed and/or treated for at least 6 months regardless of their age, sex, stage of the disease, and type of the therapy. Continuity of the out-patient clinic visits for follow-ups was confirmed, and the general status of the patients had not deteriorated. The study was planned in accordance with the Helsinki Declaration, and the approval of the Ethics Committee of the İstanbul University Cerrahpaşa Medical Faculty was obtained. Written informed consent of the patients to participate in the study was received. Participants who chose to quit the study for any reason, cases lacking any results from the analyses done for the evaluations, cases of non-continuance of follow-up visits, and patients who died before completing the follow-up period were not included in the study.

**Methods**

The melphalan and prednisone (MP; group 1); vincristine, adriamycin (doxorubicin), and dexamethasone (VAD; group 2); thalidomide (group 3); and bortezomib (group 4) regimens were as defined before [6,7,8,9]. International Myeloma Working Group response criteria were used while evaluating the patients’ responses to these treatment modalities [10]. All patients had received monthly zoledronic acid treatment, and since the serum BALP values could be affected, the patients with renal insufficiency who required dialysis were excluded from the study population.

The levels of BALP (initial, third month, and sixth month), serum protein electrophoresis, C-reactive protein, erythrocyte sedimentation rate, lactate dehydrogenase, total protein, albumin, hemoglobin, beta-2 microglobulin, creatinine, glomerular filtration rate, calcium, quantitative immunoglobulin and light chain, and immunofixation electrophoresis of all patients were analyzed in the Fikret Biyal Central Biochemistry Laboratory of the İstanbul University Cerrahpaşa Medical Faculty.

Four milliliters of peripheral blood was taken from all patients into tubes containing EDTA both prior to the treatment and at the third and sixth months of treatment. BALP levels were measured by means of radioimmunoassay technique using the Ostease BALP Kit of International Diagnostic Systems Ltd. The normal ranges of BALP levels among pre-menopausal women, post-menopausal women, and men are 11.6-29.6 U/L, 14.2-42.7 U/L, and 15.0-41.3 U/L, respectively, as given by the manufacturer.

Complete blood count was evaluated with a Beckman Coulter HMX auto-analyzer in the laboratory of the Hematology Division. Bone marrow aspiration and biopsies were evaluated in the Pathology Department of Cerrahpaşa Medical Faculty.

**Statistical Evaluation**

Statistical analyses were done with the NCSS 2007 package program. In addition to the descriptive methods (mean, standard deviation), Friedman’s test was used for the repetitive measurements of the multiple groups, the Kruskal-Wallis test was used for intergroup comparisons, Dunn’s multiple comparison test was used for the comparison of sub-groups, and chi-square and Fisher’s exact tests were used for the comparison of qualitative data. The level of statistical significance was set at p<0.05.

## RESULTS

Among the 147 patients with MM, 113 patients were eligible for evaluation. Of these 113 patients, 52 (40%) were female and the average age was 60.5 years (range: 30-84 years). The patients included in the study were either newly diagnosed (n=45) or had active relapsed disease (n=42), or were receiving no treatment (n=26) [including patients with both stage I disease (n=11) and with post-treatment remission (n=15)]. The patients who had received treatment were divided into groups receiving MP, VAD, thalidomide, and bortezomib. There were 45 newly diagnosed patients, of which 25 had received MP, whereas 20 had received VAD. The 42 active relapsed patients had received novel agents (thalidomide was given in 17 patients and bortezomib in 25 patients).

The distribution of the groups was homogeneous, and there was no statistical difference between the groups according to age or sex at p=0.134 and p=0.183, respectively. IgG was the most common type of paraproteinemia. The distribution rates of M protein were as follows: 70 patients (61.9%) had IgG, 29 patients (25.66%) had IgA, 6 patients (5.30%) had kappa light chain, and 8 patients (7.07%) had lambda light chain type disease. The majority of the patients were in the advanced stage. According to the Durie-Salmon (DS) staging system, 11 (9.7%) patients were in stage 1, 27 (23.9%) patients were in stage 2, and 75 (66.4%) patients were in stage 3. According to the International Staging System, 50 patients (44.2%) were in stage 1, 41 patients (36.3%) were in stage 2, and 22 patients (19.5%) were in stage 3. The distributions of the patients according to the myeloma types and stages are displayed in Table 1.

There was no significant difference according to the pre-treatment, third month, and sixth month mean BALP values among the MP, VAD, and thalidomide groups (p=0.069, p=0.148, p=0.254; Table 2). There was a significant change between the pre-treatment, third month, and sixth month mean BALP values in the bortezomib group (p=0.002). While the sixth month mean BALP value was significantly higher than the pre-treatment and third month mean BALP values (p=0.003), no significant difference was found between pre-treatment and third month values (p>0.05). Significant change was found between initial, third month, and sixth month mean BALP values of the no-treatment group (p=0.0001) (Table 2). The initial mean BALP values were found to be significantly lower than the third and sixth month mean BALP values (p=0.001, p=0.035). Since the patient numbers in the groups were relatively small, especially when divided according to DS stages, we did not compare BALP values among the patient groups according to the DS staging system.

## DISCUSSION

Myeloma-induced skeletal problems negatively affect the quality of life [11,12,13].

The activation of osteoclasts and the suppression of osteoblasts are the main events in MM. Bone formation is suppressed. Therefore, bone lesions are totally lytic in patients with MM [14]. Osteoclasts usually proliferate on the resorptive surfaces adjacent to the myeloma cells in the bone and do not proliferate in the regions that do not have tumoral involvement [15].

Until the new treatment options were developed, MP, high-dose dexamethasone, VAD, and multi-drug combination chemotherapies were the combinations most frequently used in MM [16,17,18,19]. The new drugs that have recently been included in the treatment of MM are molecular-targeted drugs that target the micro-environment of the bone. Among those drugs, good therapy responses have been obtained with the combination of thalidomide, bortezomib, or lenalidomide with high-dose dexamethasone [20]. The treatment of myeloma bone disease has mainly been directed toward the inhibition of osteoclastic activity with the use of bisphosphonates [11].

Anti-myeloma therapies that cause remission are usually not accompanied by an increase in the osteoblast indicators or in bone mineral density [21,22].

When compared with patients with monoclonal gammopathy of undetermined significance or with the healthy controls, deterioration in osteoblastic activity in the patients with MM was observed with a decrease in osteocalcin or BALP levels [23]. In healthy subjects, BALP accounts for approximately 50% of the total alkaline phosphatase (TAP) in the circulation. BALP reflects both the bone formation and the bone degradation more sensitively than alkaline phosphatase [24]. Serum BALP shows a remarkable correlation with the dynamic parameters of bone formation [25].

The fact that there is a relationship between the increase in serum BALP and osteoblastic activity in MM has been demonstrated in another study, as well [26]. Some preclinical trials again raised the suggestion that the inhibition of proteasome might enhance osteoblastic activity [27]. From this perspective, proteasome inhibitors represent a group of new anti-tumor drugs [28] and have strong anti-myeloma effects [29].

Bortezomib, which is a proteasome inhibitor, is the first drug used in the treatment of MM that has similar effects on the osteoblastic function together with important anti-myeloma activity [11,14,15,30,31,32,33,34]. Bortezomib, a first-generation proteasome inhibitor that has been developed to be used as an anti-neoplastic agent, inhibits the 26S proteasome that is located on the chymotryptic region and inhibits the proliferation of chemotherapy-sensitive, chemotherapy-resistant, and dexamethasone-resistant MM cells as well as the proliferation of MM cells that have been recently isolated from the bone marrow of the patients [35,36].

The first evidence concerning the effect of bortezomib on bone metabolism came from a 63-year-old female patient with κ-chain MM who had undergone sequential autologous transplantation and was then treated with 1 mg/m^2^ bortezomib on days 1, 4, 8, and 11 of a 21-day cycle. The paraprotein response of myeloma was associated with a rapid increase in TAP. The effect continued for a few therapy cycles and the BALP values were normal during the relapses [9,37]. Sezer et al. reported a negative correlation between RANKL and osteoprotegerin [38]. Subsequently, a significant increase was determined in BALP levels in the group receiving bortezomib as compared to the control group, and this finding was interpreted as osteoblastic activity increasing the effect of bortezomib. Thereafter, the change in alkaline phosphatase levels was retrospectively analyzed and evaluated in 2 large studies (APEX and SUMMIT studies) in which bortezomib was used as a single agent in patients with recurrent/refractory MM [39,40]. Data obtained from all these studies show that bortezomib increases osteoblastic activity.

In the present study, the initial, third month, and sixth month BALP levels were not significantly different in the MP, VAD, and thalidomide groups. Significant change was observed among the pre-treatment, third month, and sixth month mean BALP values of the bortezomib group. The sixth month mean BALP value was found statistically higher than the pre-treatment and third month mean BALP values.

Significant change was observed between the initial, third month, and sixth month mean BALP values of the no-treatment group. The initial mean BALP value was found statistically lower than the third and sixth month mean BALP values, whereas no statistically significant difference was observed between the other times. According to our findings, the high BALP values at the third and sixth months of therapy in the group receiving bortezomib can be interpreted as the osteoblastic activity being increased in this group.

The results of our study are also in line with the clinical findings of Shimazaki et al., who defined high BALP levels following bortezomib combination therapy in resistant MM [41]. In addition, it was reported that there was no significant change in BALP levels in patients receiving dexamethasone, whereas there was a significant increase in BALP levels in the myeloma patients receiving bortezomib [39,42]. In the present study, we determined a significant difference between the pre-treatment and post-treatment BALP values with bortezomib therapy. On the other hand, the degree of improvement obtained by chemotherapy not being in line with the healing degree of the bone disease may be related to the increased osteoclastic activity of MM even in the plateau period [21,22].

In the present study, the third and sixth month mean BALP values being high in the no-treatment group may be explained by decreased osteoclastic and increased osteoblastic activity, because the majority of the patients in this group were in remission (complete and/or almost complete). In some studies concerning MM, it was determined that BALP levels were associated with bone pain, lytic lesions, and bone fracture [43]. From this point of view, achieving complete remission and treating the bone disease appear to be important aims for decreasing the morbidity and mortality of the patients.

In conclusion, the use of proteasome inhibitors such as bortezomib, together with bisphosphonates, will no doubt lead to much more positive outcomes in myeloma treatment.

**Conflict of Interest Statement**

The authors of this paper have no conflicts of interest, including specific financial interests, relationships, and/or affiliations relevant to the subject matter or materials included.

## Figures and Tables

**Table 1:**
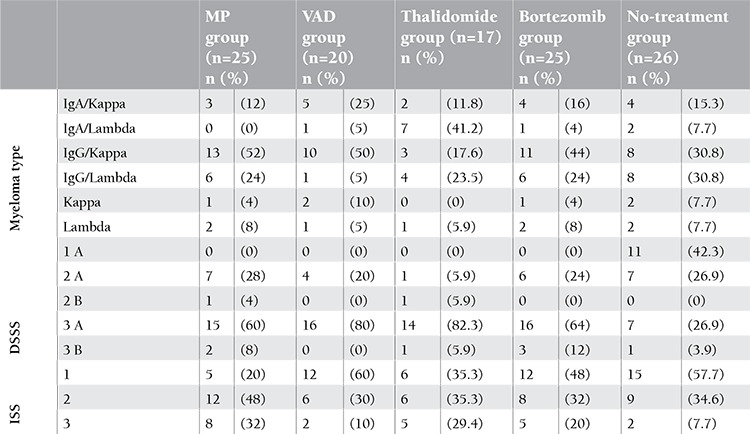
The distribution of the patients according to the myeloma types and stages (DSSS: Durie-Salmon Staging System, ISS: International Staging System, MP: melphalan and prednisone, VAD: vincristine, adriamycin, and dexamethasone).

**Table 2:**
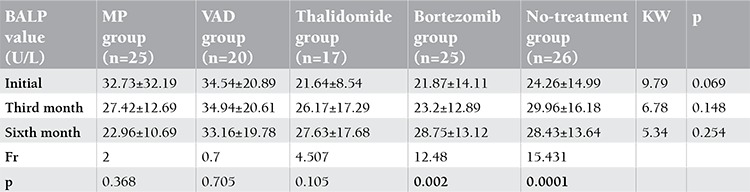
The change in BALP values among different treatment groups (BALP: bone-specific alkaline phosphatase).
